# An analysis of inhibition of the severe acute respiratory syndrome coronavirus 2 RNA-dependent RNA polymerase by zinc ion: an *in silico* approach

**DOI:** 10.2217/fvl-2020-0369

**Published:** 2021-04-26

**Authors:** Sina Zoghi, Hossein Jafari Khamirani, Seyed Alireza Dastgheib, Mehdi Dianatpour, Alireza Ghaffarieh

**Affiliations:** 1 ^1^Student Research Committee, Shiraz University of Medical Sciences, Shiraz, Iran; 2 ^2^Department of Medical Genetics, Shiraz University of Medical Sciences, Shiraz, Iran; 3 ^3^Stem Cells Technology Research Center, Shiraz University of Medical Sciences, Shiraz, Iran; 4 ^4^Massachusetts Eye & Ear Infirmary, Harvard Medical School, Boston, MA 02114, USA

**Keywords:** bioinformatics, COVID-19, *in silico*, RNA-dependent RNA polymerase, SARS-CoV-2, zinc

## Abstract

**Background:** Coronavirus disease 2019 is caused by exposure to severe acute respiratory syndrome coronavirus 2 (SARS-CoV-2). It was reported that Zn^2+^ is an inhibitor of severe acute respiratory syndrome coronavirus (SARS-CoV). We hypothesize that the same applies to the newly discovered SARS-CoV-2. **Material & methods:** We compared the structure of RNA-dependent RNA polymerase between SARS-CoV and SARS-CoV-2. The RdRp’s binding to Zn^2+^ was studied by metal ion-binding site prediction and docking server. **Results:** Several regions containing key residues were detected. The functional aspartic acid residues RdRp, 618D, 760D and 761D were among the predicted Zn^2+^-binding residues. **Conclusion:** The most probable mechanism of inhibition of RdRp by Zn^2+^ is binding to the active aspartic acid triad while other binding sites can further destabilize the enzyme or interfere with the fidelity-check mechanism.

## Tweetable abstract

The most probable mechanism of inhibition of RNA polymerase by Zn^2+^ is binding to the active aspartic acid triad while other binding sites can further destabilize the enzyme or interfere with the fidelity-check mechanism. #Zinc #SARS_CoV_2 #COVID_19.

Coronavirus disease 2019 (COVID-19) is caused by severe acute respiratory syndrome coronavirus 2 (SARS-CoV-2). SARS-CoV-2, alongside SARS-CoV and the Middle East Respiratory Syndrome coronavirus, belong to the beta-coronavirus genus. These three viruses have caused three health crises in the past decade. The last one has started in 2019 and is still ongoing on. Thus far, over 35 million COVID-19 cases and one million COVID-related deaths are recorded globally (https://coronavirus.jhu.edu/, 6 October 2020).

Although a massive number of clinical trials are being conducted all around the globe, no proven cure is available for the virus presently. Once promising medications like hydroxyl chloroquine (www.recoverytrial.net/) have been abandoned, while new candidates have emerged [1]. Roughly 30 studies are registered at clinicaltrials.gov investigating the role of zinc in combination with other drugs against COVID-19, either as a main or supplementary treatment.

Zinc ion toxicity is only reported after a high amount of daily consumption, making it extremely rare (symptoms are seldom observed for less than 1 or 2 g of ingestion.) [2,3]. Acceptable tolerability and safety at sufficiently high doses make Zn^2+^ more favorable for treatment or prophylaxis compared with common antiviral agents including nucleoside analogues.

Hitherto, inhibitory effects of zinc on several RNA viruses have been documented [4–15]. It was shown by Velthuis *et al.* [16] that zinc ion can reversibly inhibit the RNA-dependent RNA polymerase of SARS-CoV from the 2003 pandemic, dose-dependently, in both initiation and elongation phases. Due to the compelling similarities between the two viruses, one can easily infer that this phenomenon could also apply to the newly discovered SARS-CoV-2.

Zinc ion’s interaction with the RdRp of SARS-CoV-2 is examined closely in the present paper and the potential mechanism of inhibition is accordingly proposed. Application of Zn^2+^ supplementation for COVID-19 patients has been suggested elsewhere [17–19]. An *in silico* approach was chosen over the wet-lab experimental approach because of convenience as a first step.

## Materials & methods

The SARS-CoV-2 RNA-dependent-RNA-polymerase (PDB ID: 6M71) [20] was studied for zinc ion binding. Different structures including (PDB ID:7BV2) [21], (PDB ID:7BTF) [20], (PDB ID:6YYT) [22], (PDB ID:7C2K) [23] were utilized to interpret and validate the results.

### Sequence & structure comparison

Multiple sequence alignments were generated by MUSCLE [24] with the default setting. Structures were compared by ‘Matchmaker’ and ‘Match ->Align’ tools embedded in UCSF Chimera [25].

### Evolutionary conservation of amino acids

Conservation of residues from (PDB ID: 6m71) was analyzed by Consurf (https://consurf.tau.ac.il/) [26] (Supplementary Figure 2). Results were visualized by built-in NGL viewer [27].

### Molecular docking & interaction studies

MIB: metal ion-binding site prediction and docking server’s online server (http://bioinfo.cmu.edu.tw/MIB/) [28,29] was used to predict the binding sites and residues in SARS-CoV-2’s RdRp. For the analysis, NSP12 from (PDB ID: 6m71) and NSP8 and NSP7 from (PDB ID: 7BTF) (due to imperfections in NSP8, chain B, of 6M71 structure) were separately uploaded to MIB online server.

### Molecular dynamics simulation

The flexibility of the segments of RdRp was simulated by calculating root-mean-square fluctuations of the residues utilizing CABS-flex 2.0 online web server. The analysis resulted in a score attributed to each residue and a fluctuation map of the whole protein. The data were depicted as a graph by Microsoft Excel 2016 (Microsoft, WA, USA).

### Surface charge density calculation

Surface charge density calculation was carried out by DelPhi web server v2 [30–33]. Delphi calculates the electrostatic potential/energy of a biomolecule by solving the Poisson–Boltzmann equation.

## Results

The comparison between the SARS-CoV and SARS-CoV-2 RdRp shows striking similarities, in terms of both sequence and structure. Chain A (PDB ID: 7BTF) of SARS-CoV-2 was compared with its homologous protein, Chain A of SARS-CoV (PDB ID: 6NUR). Sequences retrieved from these two structures were subsequently aligned by MUSCLE. About 96.97% identity between the amino acid sequences was obtained. Structure comparison came up with overall root-mean-square deviation: 0.535, structural distance measure (SDM; cut-off set at 3.5 Å): 15.232 and Q-score: 0.903, showing high degrees of similarity.

In previous studies, it was showed that Zn^2+^, in relevant concentrations, can inhibit the RdRp of the HCV and SARS-CoV [16]. Both SARS-CoV-2 and HCV are positive-sense viruses. HCV RNA virus shares a high sequence and structural homology with SARS-CoV [20].

### Zinc-binding analysis

Zinc-binding analysis was carried out by MIB. Fe^2+^ and Cu^2+^ were studied as negative controls. Mg^2+^ binding was also analyzed for further interpretation. Each chain from (PDB ID: 6m71) was separated from the structure by UCSF chimera and studied individually. The output was uploaded to MIB online server. The sites containing zinc ion as cofactors in 7btf structure were among the top predictions by MIB (first and seventh, scores 2.126 and 1.454, respectively) showing the accuracy and reliability of the tool. All predicted values surpassing two were recognized as potential zinc ion binding sites.

The average score of docking for zinc is considerably greater than those of Fe^2+^ and Cu^2+^, revealing the greater tendency of zinc to bind to this specific protein. The average potential docking score of the top ten sites of RdRp for Fe^2+^ and Cu^2+^ ions are 1.2168 and 1.1279, respectively, compared with 1.6035 for Zn^2+^ ion. One can infer that RdRp is more geared toward binding to zinc ion rather than other divalent metal ions. As a further matter of clarification, predicted binding sites for these ions are not nearly as crucial as those of zinc ion. As an example, the three main aspartic acid residues, D618, D760 and D761, are not among the predicted sites for the other two divalent metal ion, while zinc ion can bind to these residues and potentially alter the function of the region.

### NSP12

The 269D and 271L are closely associated with NSP8 (108-NADG-113) loop. The 269D and 271L are predicted to be associated with Zn^2+^ which appears to interrupt the interaction of the two chains and destabilize the whole protein. The 272K and 278E, located on the same loop, are also predicted to bind to Zn^2+^.

M380 and H381 residues, located in the fingers subdomain (residues L366-A581 and K621-G679) [20], are highly likely to bind to zinc ion (prediction score 5.043 and docking score 1.903) ranked second only to the first allosteric site (295H, 301C, 306C and 310C residues). These residues are situated close to the first alpha-helix of NSP8 chain B (90-MLFTM-94) and NSP12 residues including V341. Distortion in the structure or binding of NSP12 and NSP8, arising from the introduction of Zn^2+^ is expected.

The 477D and 481D residues (prediction score: 2.611, docking score: 1.041) can interfere with the proper function of RdRp due to proximity to F480. Although these two residues are neither located in the catalytic site nor substrate-binding pocket, they are crucial to nucleotide fidelity-check preceding the incorporation [34,35].

D760 and 761, located in the palm subdomain, are essential to the function of the RdRp. The MIB output indicated that these residues are potential binding sites for zinc ion (prediction score: 2.51, docking score: 1.030) [22,36].

N857 in PDB ID: 7BZF is involved in a hydrogen bond with the G9 base from the RNA double helix (www.ncbi.nlm.nih.gov/Structure/cdd/cdd.shtml) [37]. Furthermore, T853 is interacting with Y71 from NSP8 (7C2K). In other available structures, these residues and their surrounding region are also closely related to the RNA double helix (Supplementary Figure 4).

### NSP7 & NSP8

Zinc ion also binds to two aspartic acid residues, 161D and 163D, of NSP8 (score: 6.117 in chain B PDB ID: 6M71 and 7.141 in chain D PDB ID: 7BTF). These two residues from NSP8 Chain B are directly interacting with SER 26 from NSP7 (PDB ID: 7BTF), the only interaction between NSP7 and NSP8 Chain B. However, the same residues are outwardly directed in the NSP8 Chain D. These residues are also situated near P183 in both B and D Chains, which could disrupt the interactions and alter the spatial arrangement of the region. 161D can form another interaction with Zn^2+^ when accompanied by 171E (docking score: 0.967, prediction score: 5.502). Their sidechains and the predicted Zn^2+^ are near P183. The 99D and 101D (docking score: 0.734, prediction score: 4.130) are predicted to interact with Zn^2+^. 99D and 101D are also closely situated near 338V and 339P. No noteworthy binding site in NSP7 was detected (Supplementary Figure 3).

## Discussion

Theoretical evidence supporting Zn^2+^ efficacy against SARS-CoV-2 and the relatively low adverse events tied to high zinc intake provides an exceptional opportunity for fighting COVID-19. With the number of dead over one million people, few signs of relief in the near future are noticeable [38]. According to previous findings and our results, zinc ion effect on the RdRp would probably show a dose-dependent fashion; minimal activity at zero concentration, rising to the highest in the optimal concentration (supposedly the physiologic concentration in the host cell) and finally falling as the new ions are introduced to the environment.

It is well proven elsewhere that the metal ions can alter the physical properties of proteins, namely thermal stability and conformational rigidity/flexibility [33,39,40]. It is reported that zinc ion, through binding, can decrease the thermal stability and rigidity of calreticulin, causing a structural rearrangement and intensifying the hydrophobic character of the protein and paving the way for more self-association [39].

One way of altering the function and affinity of the enzymes for the substrates is to reduce the flexibility of the key regions, which can even lead to spatial blockade of the substrate-binding sites either by changing the intrinsic features of the protein or alteration of the environment properties. Protein flexibility allows for increased affinity for the substrate [41–43]. Zn^2+^ can conceivably interfere with the normal functionality of the RdRp by rigidifying the flexible binding regions or destabilizing and occupying key inter- or intra-peptide interactions and binding sites (Figure 2).

**Figure 1.**
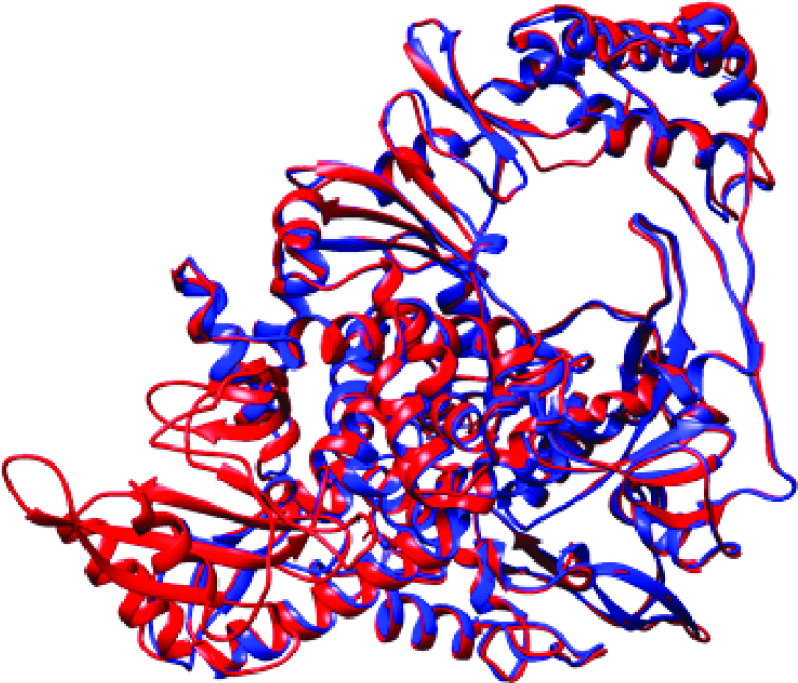
RNA-dependent RNA polymerase of severe acute respiratory syndrome coronavirus superimposed on RNA polymerase of severe acute respiratory syndrome coronavirus 2. Red: NSP12-7BTF, blue: NSP12-6NUR. RdRp: RNA polymerase; SARS-CoV: Severe acute respiratory syndrome coronavirus; SARS-CoV-2: Severe acute respiratory syndrome coronavirus 2.

The 269D, 271L, 272K are on the hindmost part of (257-VDTDLTKPYIKWDLLKYD-274) loop while 278E is on the neighboring α-helix. This loop is in close proximity to NSP8 (109-NARDG-113) loop (conservation scores respectively: 7, 9, 5, 5 and 7). All residues except 278E are among putative NSP8 binding sites of the NSP12 (www.ncbi.nlm.nih.gov/Structure/cdd/cdd.shtml) and are relatively flexible (Figure 1, Supplementary Figure 1A & B) which allows them to have such interactions. Zn^2+^ binding can both change the conformation of the side chains and decrease the flexibly of the loop by direct interactions with these residues; thus, possibly changing the interrelation of these two loops and, by extension, that of NSP12 and NSP8. The 380M and 381H go down the same path. Although 380M and 381H are not as flexible as mentioned residues, they are located in proximate with the first alpha-helix of NSP8 Chain B (90-MLFTM-94) (conservation scores respectively: 7, 9, 8, 5 and 9). Zn^2+^ binding to M380 and H381 would put it in close quarters with NSP8 residues, like previously mentioned residues, disrupting the interrelations of the two chains. Possible disruption of the bond between 380M and V341 can increase the flexibility, paving the way for greater distortion in this region. The above-mentioned residues on NSP12 are not highly conserved while the interacting loops from NSP8 are estimated to be well conserved, indicating the potential importance of targeting these residues. The 99D and 101D of NSP8 are also predicted to interact with Zn^2+^ while closely situated to 338V and 339P of NSP12 (conservation scores respectively: 9 and 1). The predicted positioning of Zn^2+^ can also effectively interfere with the attachment of the NSP8 and NSP12 in this specific site.

**Figure 2.**
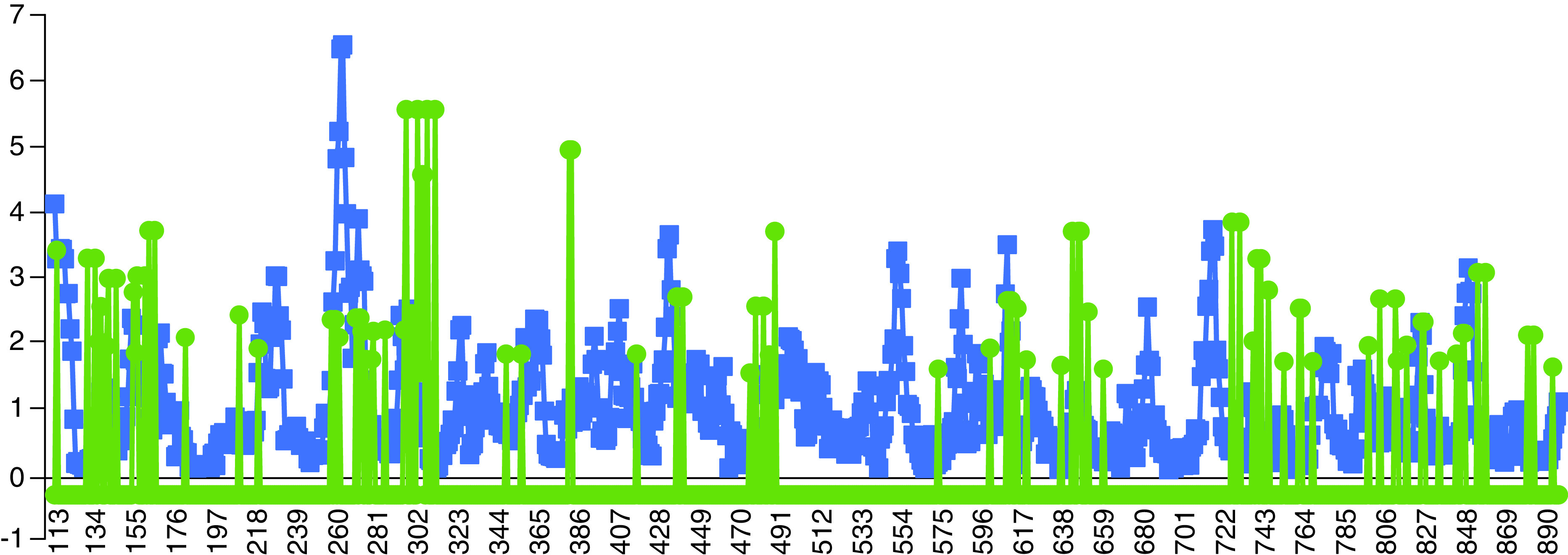
Flexibility of each residue against its binding affinity. Blue represents the flexibility (root-mean-square deviation) attributed to each residue by CABS-flex 2.0. Green represents the binding affinity of each residue generated by metal ion-binding site prediction and docking server.

R80 (conservation score: 8) from NSP8 is an interaction point with the downstream base-paired template in Chain D. D78 and K82 (conservation score for both residues: 8) are among predicted binding sites for Zn^2+^ (7BTF chain B prediction score: 2.783 docking score: 0.717). However, R80 and phosphate interaction will not be affected by Zn^2+^. It stems from the structure of the alpha-helix in the region forcing the side chains of D78 and K82 to face to the opposite side of that of R80 (72CK).

A 183P is essential for interaction with NSP12 and its substitution is lethal to the virus [36]. The 161D, 163D and 171E are NSP8’s residues predicted to bind Zn^2+^. Their closeness to 183P (conservation score: 9) can change the positioning of 183P and its sidechain, probably altering its function in both NSP8 molecules. A 163D in NSP8 (B) is also interacting with 26S in NSP7, the only direct interaction between the two molecules, which can be negatively impacted by the Zn^2+^ binding. All of these residues are highly conserved, showing their importance in maintaining the protein’s overall integrity.

Although F480 is neither located in the catalytic site nor substrate-binding pocket, it is crucial to nucleotide fidelity-check, preceding the incorporation of the nucleosides to the chain alongside V557L in SARS-CoV [34,35]. Shannon *et al.* and Agostini *et al.* presented the importance of this residue in inhibition of the RdRp by Remdesivir. The substitution of this residue by leucine confers resistance to Remdesivir at the cost of the *in vitro* activity of the enzyme; resulting in imperfections in the enzyme’s function. The 477D and 481D as stated before, are predicted to bind to Zn^2+^, which can adversely affect the function of the neighboring F480 (conservation score:9).

D618, D760 and D761 are believed to be the canonical aspartic acid residues responsible for the coordination of the divalent cations. D618, located in motif A, is the divalent cation binding residue, conserved (conservation score: 9) among HCV ns5b, poliovirus 3Dpol and coronaviruses. D760 and D761 are located in motif C, forming the conserved (Table 1) catalytic residues (759-SDD−761), analogous to 317-GDD−319 in HCV ns5b and 327-GDD−329 poliovirus’ 3Dpol [20,44–46]. These residues have also been linked to inhibition of RdRp by Remdesivir and Sofosbuvir [47,48].

**Table 1.** Potential Zn^2+^ binding residues.

Chain	Residues	Template	Docking score	Prediction score	Conservation score (out of 9)
NSP12	269D, 271L	1cnqA0	0.977	2.430	6, 1
NSP12	272K, 278E	1mxdA2	0.901	2.218	1, 5
NSP12	380M, 381H	1fioA0	1.903	5.043	6, 4
NSP12	477D, 481D	1f35A3	1.041	2.611	1, 1
NSP12	760D, 761D	1bawA0	1.030	2.51	9, 9
NSP12	853T, 857E	7mdhB4	1.227	3.136	7, 9
NSP8 Chain B	161D, 163D	1no5B0	0.657	(5.502, 3.671, respectively)	8, 9
NSP8 Chain B	161D, 171E	1r4vA2	0.967	5.502	8, 5
NSP8 Chain B (chain D)	99D, 101D	1f2oA0 (1mxdA1)	0.734 (0.667)	4.130 (4.528)	9, 8

D618 is also listed as one of the key players in the enzyme’s active site (7BV2) [49]. It was not predicted to be a potential binding residue for Zn^2+^ in PDB ID: 6M71. However, in analyzing PDB ID: 7BTF, D618 was among the predicted binding sites (predicted score: 2.592) showing a possible interference with the normal function of RdRp in one of the most important functional sites of the enzyme.

Inhibition of the nidovirus’ RdRp by Zn^2+^ was even observed at low concentrations despite the coNSPicuous presence of Mg^2+^ (more than 25-fold excess) indicating that either the affinity of the active site for Zn^2+^ is much higher compared with Mg^2+^ or that Zn^2+^ does not compete with Mg^2+^ for the same binding sites and binds to other zinc-specific binding sites in the enzyme; including those interfering with protein folding, interactions of the NSP12 and its co-factors and D618, D760 and D761. MIB prediction for Zn^2+^ ion was more robust compared with Mg^2+^ ion which is present at the enzyme’s functional site (D760, D761 7BV2; scores for top ten docking sites 1.6035 and 1.5068, respectively). Most crucial, although a set of Mg^2+^ binding residues are located close to D618, D760 and D761 (600–800 residues), they do not interfere with the binding of Zn^2+^ to D618, D760 and D761. The alterations in the general stability of the protein can also be the way by which the Zn^2+^ inhibits the RdRp [50]. Zn^2+^ binding to the aspartic acid triad seems to be the most probable mechanism of inhibition of RdRp.

Zn^2+^ binding sites are typically dominated by Cys, HIis and to a lesser extent acidic residues including aspartic acid residues (www.zincbind.net/) [51]). In the present protein, however, the predicted Cys and HIis residues are less common and are either located in the main allosteric sites or too far from the active site to cause any remarkable alteration. Zinc ion does not interact with those residues participating in the stabilization of the phosphate backbone of the RNA double helix; probably as a result of their positively charged nature (Supplementary Figure 1).

## Conclusion

Zn^2+^ binding to the key interacting sites of the different chains of the RdRp, can be linked to the RdRp’s inhibition. Binding to crucial residues of the RdRp including the central aspartic acid triad could potentially block, slow down, or even negatively affect the fidelity of the replications of the virus. Additionally, binding to the key interacting sites between the NSP12 and its cofactors can insulate the chains in the first place or disrupt the overall structural integrity of the protein. Therefore, we suggest that further clinical studies are needed to confirm the role of Zn^2+^ in prophylaxis or treatment of the COVID-19.

**Summary points**

- The RNA polymerase is identical between severe acute respiratory syndrome coronavirus and severe acute respiratory syndrome coronavirus 2.
- The aspartic acid triad of the active site of the RNA polymerase is among the predicted binding site of the zinc ion.
- The zinc ion can interfere with interrelation of the polypeptides, fidelity-check mechanism or the active site of NSP12.
