# Supplementary material for: An analysis of inhibition of the severe acute respiratory syndrome coronavirus 2 RNA-dependent RNA polymerase by zinc ion: an in silico approach
Source: Future Virol. 2021 Apr 26:10.2217/fvl-2020-0369. doi: 10.2217/fvl-2020-0369 (PMC8074572; doi:10.2217/fvl-2020-0369)
Supplement: Supplementary file 1 [file supplementary_material.docx]

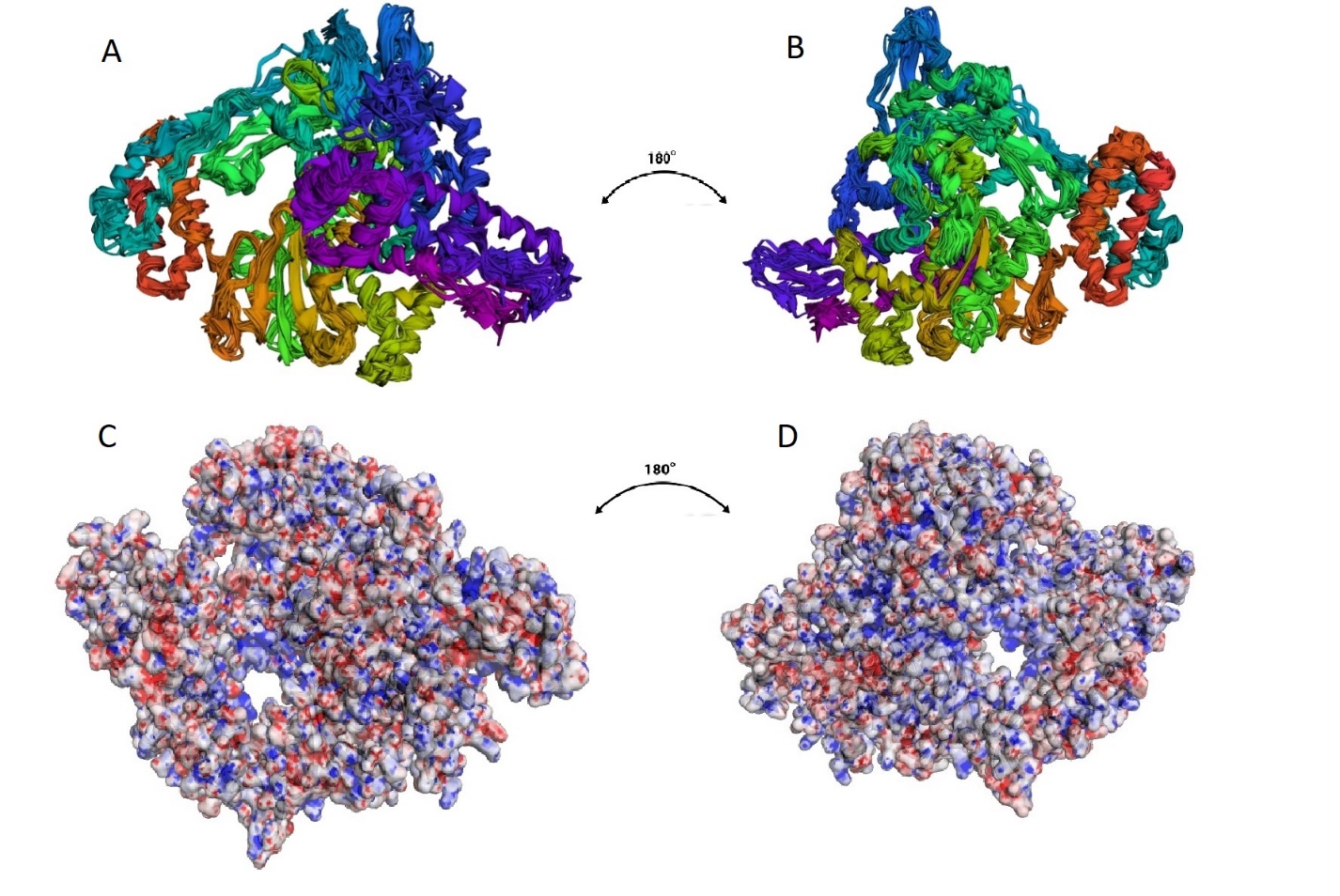


Supplementary Fig 1. A and B. conformational models of SARS-CoV-2 RdRp, C and D. surface charge density of SARS-CoV-2 RdRp


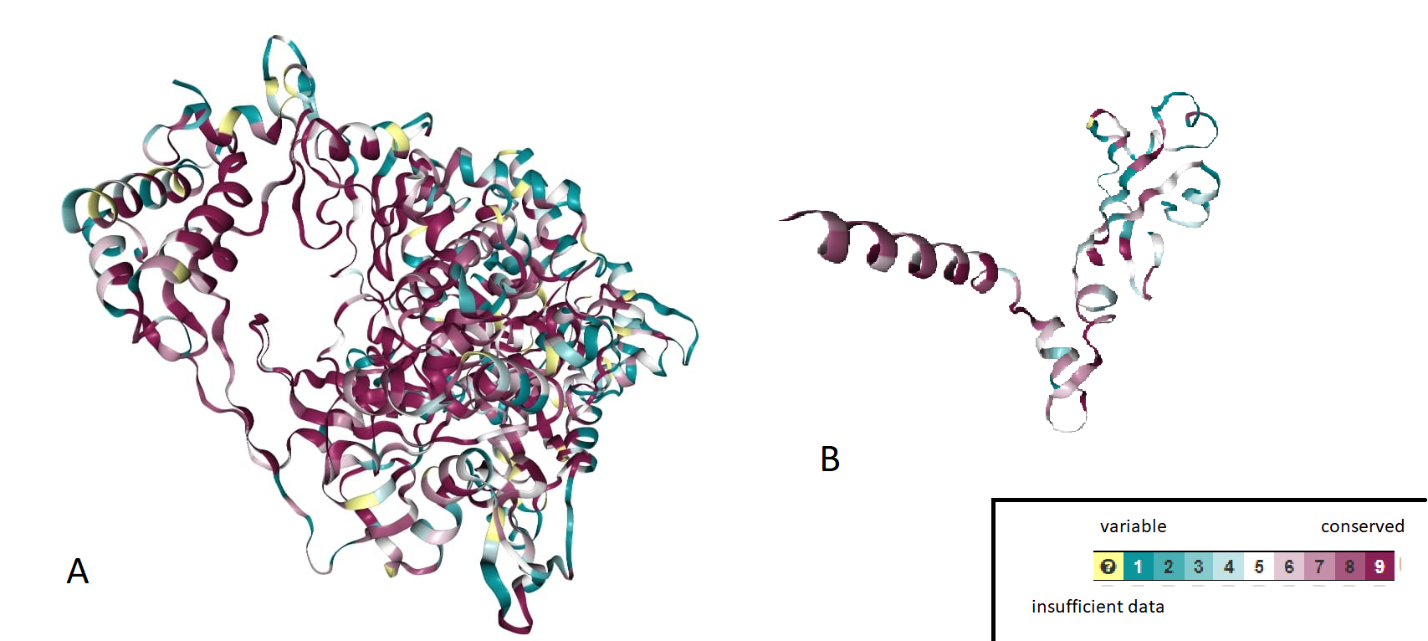


Supplementary Fig 2. Conservation of residues of SARS-CoV-2 RdRp. A. NSP12, B. NSP8


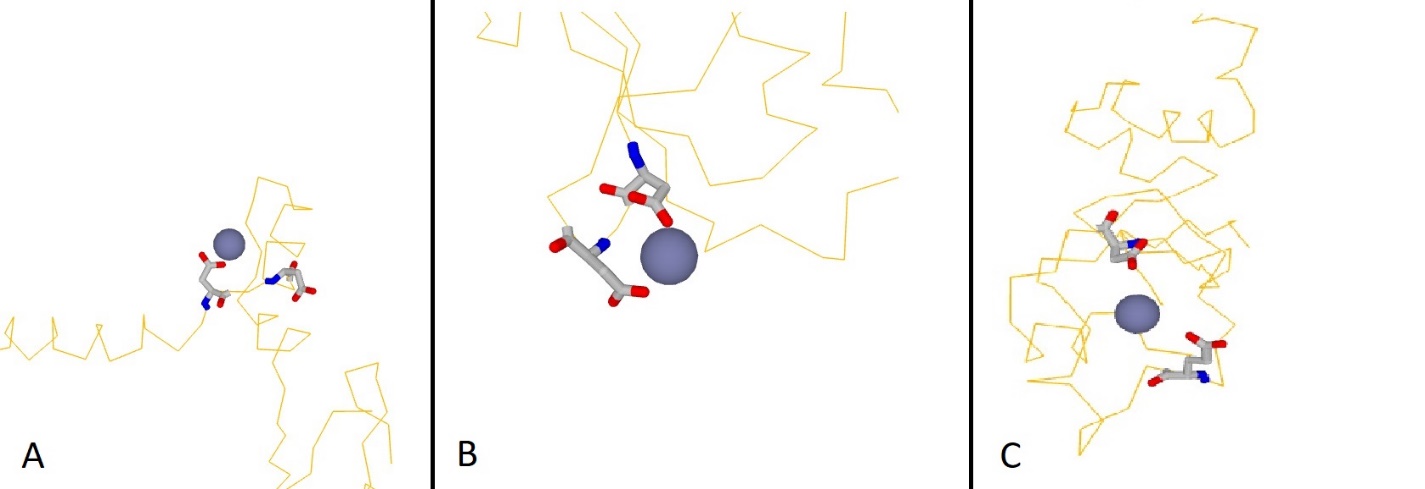


Supplementary Fig 3. Positioning of Zn^2+^ close to its predicted binding NSP8 residues. A. 99D and 101D, B. 161 and 163, C. 161D and 171E


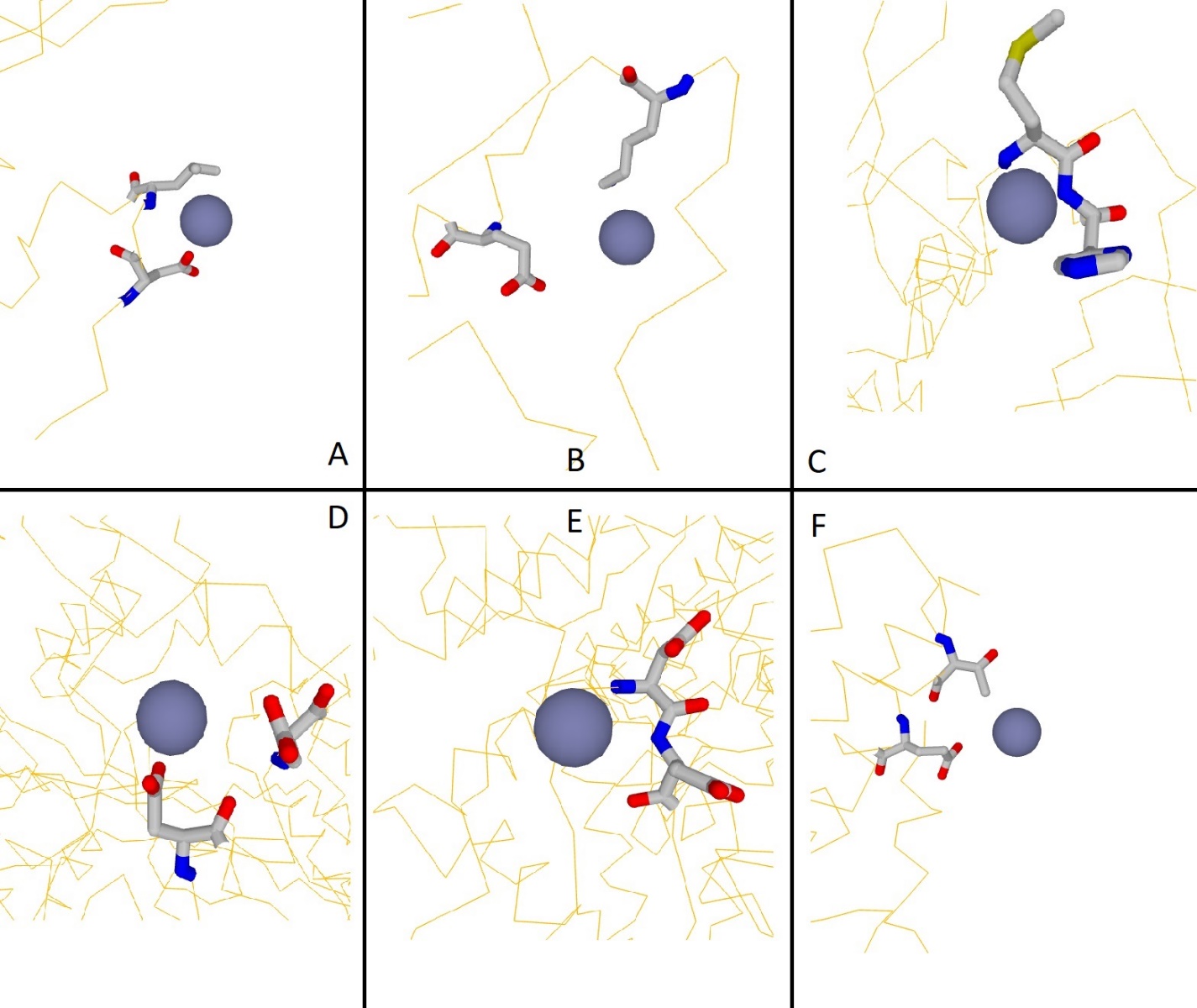


Supplementary Fig 4. Positioning of Zn^2+^ close to its predicted binding NSP12 residues. A. 269D and 271L, B. 272K and 278E, C. 380M and 381M, D. 477D and 481D, E. 760D and 761D, F. 853T and 857E
